# Investigating the Impact of Cannabis Consumption on Hospital Outcomes in Patients With Primary Spontaneous Pneumothorax: A Nationwide Analysis

**DOI:** 10.7759/cureus.55601

**Published:** 2024-03-05

**Authors:** Aman Goyal, Mohammed A Quazi, Rayika Syed, Hafiz Abdullah Ikram, Farooq A Sheikh, Asif Farooq, Sulaiman Sultan, Abu Baker Sheikh

**Affiliations:** 1 Department of Internal Medicine, Seth Gordhandas Sunderdas Medical College and King Edward Memorial Hospital, Mumbai, IND; 2 Department of Psychiatry and Behavioral Sciences, University of New Mexico School of Medicine, Albuquerque, USA; 3 Department of Internal Medicine, University of New Mexico School of Medicine, Albuquerque, USA; 4 Department of Family and Community Medicine, Texas Tech University Health Sciences Center, Lubbock, USA; 5 Department of Nephrology, University of New Mexico School of Medicine, Albuquerque, USA

**Keywords:** national inpatient sample, critical care medicine, pulmonology, spontaneous pneumothorax, cannabis

## Abstract

Introduction

Existing data suggest an association between primary spontaneous pneumothorax (PSP) and cannabis consumption, although evidence remains controversial.

Methods

This study used the 2016-2019 National Inpatient Sample Database to examine inpatients with PSP, categorizing them as cannabis users and non-users. Multivariate regression analyzed continuous variables, chi-square assessed categorical variables, and logistic regression models were built. Propensity score matching (PSM) mitigated the confounding bias.

Results

A total of 399,495 patients with PSP were admitted during the study period (13,415 cannabis users and 386,080 non-cannabis users). Cannabis users were more likely to be younger (p<0.001) and male (p<0.001) with a lower risk of baseline comorbidities than non-users. Cannabis users had a lower risk of sudden cardiac arrest, vasopressor use, the development of acute kidney injury, venous thromboembolism, the requirement for invasive and non-invasive mechanical ventilation, hemodialysis, ventilator-associated pneumonia, and the need for a tracheostomy. Cannabis use was associated with a 3.4 days shorter hospital stay (p<0.001), as confirmed by PSM analysis (2.3 days shorter, p<0.001). Additionally, cannabis users showed a lower risk of in-hospital mortality (p<0.001), a trend maintained in the PSM analysis (p<0.001).

Conclusions

Our study revealed correlations suggesting that cannabis users with PSP might experience lower in-hospital mortality and fewer complications than non-cannabis users.

## Introduction

Pneumothorax refers to the accumulation of air in the pleural space. Spontaneous pneumothorax is a type that occurs without any external events, such as trauma [1]. It is further divided into two subtypes: primary spontaneous pneumothorax (PSP) and secondary spontaneous pneumothorax (SSP). PSP affects individuals who have no previously diagnosed lung conditions, while SSP is associated with existing lung pathology [2,3]. The annual incidence of spontaneous pneumothorax is higher in males, with 17-24 per 100,000 men and one to six per 100,000 women. PSP occurs more often in younger individuals, while SSP occurs in the elderly [4-8].

Tobacco smoking is a significant risk factor that has been linked to a heightened risk of developing PSP. Studies have shown that smokers are at a substantially greater risk of developing health problems compared to non-smokers, with the risk increasing up to 20-fold [9,10]. With appropriate medical intervention, including needle aspiration, chest tube placement, or surgery in persistent cases, patient recovery is highly probable. 

Mortality rates associated with cannabis-induced pneumothorax are not well-documented in the literature. Some studies suggest a mortality rate ranging from 1% to 15% in patients with pneumothorax, making it an important pathological condition that warrants further research [1,11]. Emerging data suggest a link between PSP and cannabis consumption, but this association remains underexplored [9,10]. Our study aims to compare demographic information, comorbidities, hospital complications, outcomes, and quality of care metrics between PSP patients who do and do not use cannabis. A particular focus of our analysis is the mortality associated with cannabis-induced pneumothorax, which has not been sufficiently addressed in the literature.

## Materials and methods

This study utilized data from the National Inpatient Sample (NIS) database from 2016 to 2019. Since the NIS data were anonymized and publicly available, Institutional Review Board approval was not required for this study. We specifically identified and extracted data on patients with PSP and categorized them into cannabis users and non-users.

Multivariate regression models were employed to analyze continuous variables, while chi-square tests for independence were applied to categorical variables. To standardize hospital costs from 2016 to 2019, the Consumer Price Index Python package was utilized. Logistic regression models were constructed to derive odds ratios (OR) for the variables of interest between the two cohorts.

Furthermore, predictors of mortality were explored using the Cox Proportional Hazards model, exclusively for the cannabis cohort. Subsequently, a propensity score-matched (PSM) sample was generated by considering Elixhauser comorbidities and the available demographic variables for both cohorts. The same analyses were repeated for the matched cohorts. The analytical procedures and statistical models used were Python (Python Software Foundation, Wilmington, USA) R Core Team (R Foundation for Statistical Computing, Vienna), and SAS 9.4 software (SAS Institute Inc., Cary, NC).

## Results

Our study included 399,495 patients diagnosed with PSP, including 386,080 non-cannabis users and 13,415 cannabis users.

Demographic distribution

The mean age of males among non-cannabis users was 59.93±19.2 years, while for females it was 62.86±17.5 years. Among cannabis users, the mean age for males and females was 36.4±15.5 years and 40.4±15.5 years, respectively.

The majority of non-cannabis users were >50 years old (50-69 years, 39.0%; ≥70 years, 37.7%). By contrast, among cannabis users, the largest age group was 18-29 years (43.8%) (p<0.001, Table 1). There was a significant association between gender and cannabis consumption (p<0.001), with 60.4% of males among non-cannabis users and 81.5% of males among cannabis users.

Among non-cannabis users, 73.0% (n=281,860) were white, whereas only 60.5% (n=8120) were white (p<0.001) (Table 1). A higher percentage of cannabis users had a median household income of less than or equal to $49,999 as opposed to non-cannabis users (37.9% vs. 29.2%, respectively, p<0.001). Among cannabis users, Medicaid was the most common insurance status among patients, seen in 35.7% of the patients, whereas Medicare formed the majority of the patients among non-cannabis users, with 52.0% of the patients (p<0.001). The details of these are depicted in Table 1.

**Table 1:** Demographic information and patient characteristics comparing patients diagnosed with primary spontaneous pneumothorax who do and do not use cannabis.

Variable	Patients without cannabis use		Patients with cannabis use		P-value
	N	%	N	%	
Total n=399,495	386,080	96.64	13,415	3.36	
Gender	N	%	N	%	<0.001
Female	152,820	39.58	2480	18.49	
Male	233,260	60.42	10,935	81.51	
Mean age in each gender group	Mean	SD	Mean	SD	
Female	62.86	17.46	40.39	15.49	
Male	59.93	19.17	36.41	15.45	
Age groups in years	N	%	N	%	<0.001
18-29	36,550	9.47	5875	43.79	
30-49	53,485	13.85	4120	30.71	
50-69	150,500	38.98	3150	23.48	
≥70	145,545	37.7	270	2.01	
Race	N	%	N	%	<0.001
Asian or Pacific Islander	13,305	3.45	155	1.16	
Black	47,560	12.32	3325	24.79	
Hispanic	30,415	7.88	1390	10.36	
Native American	2025	0.52	110	0.82	
Other	10,915	2.83	315	2.35	
White	281,860	73.01	8120	60.53	
Median household income (in USD)	N	%	N	%	<0.001
≤49,999	112,650	29.18	5085	37.91	
50K-64,999	101,270	26.23	3685	27.47	
65K-85,999	92,505	23.96	2880	21.47	
≥86k	79,655	20.63	1765	13.16	
Insurance status	N	%	N	%	<0.001
Medicaid	50,200	13	4790	35.71	
Medicare	200,595	51.96	1740	12.97	
No charge	1315	0.34	185	1.38	
Other	11,950	3.1	395	2.94	
Private insurance	106,610	27.61	4165	31.05	
Self-pay	15,410	3.99	2140	15.95	

Baseline comorbidities

Cannabis users had a higher prevalence of drug abuse, smoking, alcohol consumption, depression, and AIDS (p<0.001 for all). In contrast, the baseline prevalence of coronary artery disease, myocardial infarction, hypertension, diabetes, cancer, obesity, chronic pulmonary disease, peripheral vascular disease, hypothyroidism, autoimmune disease, dementia, and chronic kidney disease was higher in non-cannabis users (p<0.001 for all). The results are presented in Table 2.

**Table 2:** Baseline comorbidities in patients diagnosed with primary spontaneous pneumothorax who do and do not use cannabis (non-propensity matched).

Variable	Patients without cannabis use		Patients with cannabis use		P-value
Comorbidities	Number	%	Number	%	
Coronary artery disease	86,935	22.52	915	6.82	<0.001
Myocardial infarction	21,180	5.49	350	2.61	<0.001
Hypertension	212,660	55.08	3175	23.67	<0.001
Diabetes	82,535	21.38	820	6.11	<0.001
Cancer	91,185	23.62	1130	8.42	<0.001
Obesity	38,595	10	660	4.92	<0.001
Drug Abuse	11,830	3.06	6050	45.1	<0.001
Smoking	182,355	47.23	9335	69.59	<0.001
Alcohol	18,250	4.73	1650	12.3	<0.001
Chronic pulmonary disease	163,680	42.4	5655	42.15	0.578
Peripheral vascular disease	32,000	8.29	455	3.39	<0.001
Hypothyroidism	40,970	10.61	265	1.98	<0.001
Autoimmune disease	13,205	3.42	145	1.08	<0.001
Depression	40,225	10.42	1500	11.18	0.004
AIDS	3435	0.89	290	2.16	<0.001
Dementia	17,620	4.56	45	0.34	<0.001
Chronic kidney disease	22,850	5.92	250	1.86	<0.001

In-hospital complications and hospital course

Cannabis users had a lower risk of sudden cardiac arrest (OR: 0.5; 95% CI: 0.4-0.7; p<0.001), vasopressor use (OR: 0.5; 95% CI: 0.4-0.7; p<0.001), development of acute kidney injury (OR: 0.7; 95% CI: 0.6-0.8; p<0.001), venous thromboembolism (VTE) (OR: 0.5; 95% CI: 0.4-0.6; p<0.001), requirement for invasive (OR: 0.5; 95% CI: 0.5-0.6; p<0.001) and non-invasive mechanical ventilation (OR: 0.6; 95% CI: 0.4-0.7; p<0.001), need for hemodialysis (OR: 0.5; 95% CI: 0.4-0.7; p<0.001), ventilator-associated pneumonia (OR: 0.4; 95% CI: 0.2-0.7; p<0.001), and need for tracheostomy (OR: 0.3; 95% CI: 0.2-0.4; p<0.001). However, the need for chest tube placement was higher among the cannabis users (OR: 1.2; 95% CI: 1.1-1.3; p<0.001). The risk of hemothorax was comparable between the two groups (OR: 0.9; 95% CI: 0.7-1.3; p=0.681) (Table 3).

**Table 3:** Complications in patients diagnosed with primary spontaneous pneumothorax who do and do not use cannabis (non-propensity matched).

Complications	Number	%	Number	%	Odds ratio	95% CI lower limit	95% CI upper limit	P-value
Sudden cardiac arrest	21,355	5.53	310	2.31	0.52	0.4	0.67	<0.001
Vasopressor use	16,005	4.15	230	1.71	0.51	0.38	0.69	<0.001
Acute kidney injury	85,720	22.2	1605	11.96	0.73	0.65	0.83	<0.001
Invasive mechanical ventilation	90,080	23.33	1825	13.6	0.52	0.47	0.59	<0.001
Non-invasive mechanical ventilation	20,505	5.31	245	1.83	0.56	0.42	0.74	<0.001
Hemodialysis	16,475	4.27	230	1.71	0.51	0.37	0.68	<0.001
Venous thromboembolism	22,950	5.94	415	3.09	0.51	0.4	0.63	<0.001
Hemothorax	7240	1.88	250	1.86	0.94	0.7	1.26	0.681
Ventilator-associated pneumonia	3260	0.84	60	0.45	0.37	0.21	0.66	<0.001
Chest tube placement	211,050	54.66	8585	64	1.19	1.1	1.3	<0.001
Tracheostomy	16,725	4.33	235	1.75	0.32	0.24	0.43	<0.001

In-hospital mortality

The rate of in-hospital mortality was lower among cannabis users than among non--cannabis users (3.5% vs. 12.7%, respectively; OR: 0.4; 95% CI: 0.3-0.5; p<0.001). The p-trend for mortality was 0.999, indicating no significant trend in mortality over the five-year period.

Predictors of mortality among cannabis users

**Figure 1:**
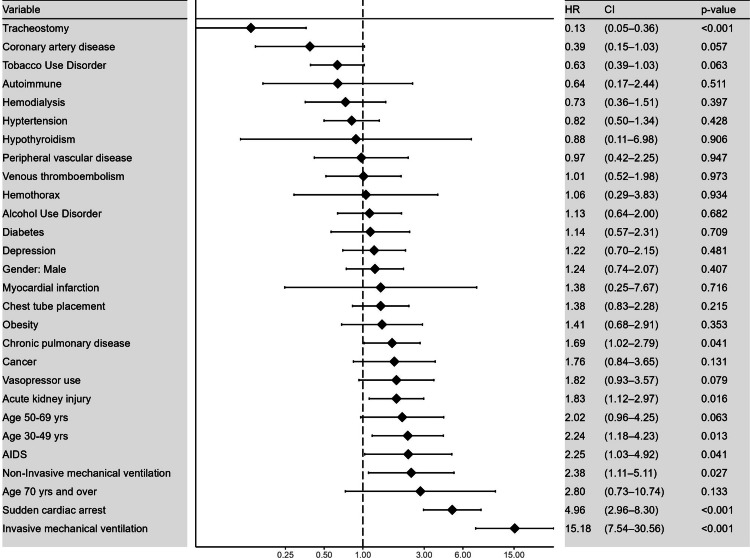
Forest plot depicting the analysis of outcomes that predicted mortality in patients who were diagnosed with primary spontaneous pneumothorax and used cannabis.

The only factor that significantly decreased the risk of mortality among cannabis users was undergoing tracheostomy (HR: 0.1; 95% CI: 0.05-0.4; p<0.001). Factors that significantly increased the risk of mortality were the presence of baseline chronic pulmonary disease (HR: 1.7; 95% CI: 1.02-2.8; p=0.04), development of acute kidney injury in the hospital course (HR: 1.8; 95% CI: 1.1-3; p=0.02), 30-49 years age group (HR: 2.2; 95% CI: 1.2-4.2; p=0.01), baseline AIDS (HR: 2.2; 95% CI:1.03-4.9; p=0.04), requirement for non-invasive mechanical ventilation (HR:2.4; 95% CI:1.1-5.11; p=0.03) and invasive mechanical ventilation (HR:15.2; 95% CI: 7.5-30.6; p≤0.001), and the development of sudden cardiac death as a complication (HR: 5; 95% CI:3-8.3; p≤0.001). The findings are presented in Figure 1.

Utilization of healthcare

The mean length of stay in the hospital among cannabis users was 8.29 days, as opposed to 10.72 days among non-cannabis users (p<0.001). After adjusting for confounding variables, the length of stay was 3.4 days shorter among cannabis users (p<0.001). From an economic perspective, the mean inflation-adjusted cost was lower among cannabis users than non-cannabis users ($26,142.3 vs. $39,538.1, p<0.001). After adjusting for confounding variables, the inflation-adjusted cost was $17,071.6 lower in the cannabis users (p<0.001). 

Analysis after PSM between the two groups

The baseline demographic and patient characteristics are presented in Table 3. About 82.1% (n=11015) of the patients who were non-cannabis users were male, as opposed to 81.5% (n=10935) among cannabis users, although there was no statistical difference (p=0.205). The largest proportion of patients in both the non-cannabis user and cannabis user groups was 18-29 years old, with 44.9% and 43.8% patients, respectively (p<0.001). Table 4 outlines the demographic information and patient characteristics of the two groups after the PSM.

**Table 4:** Propensity score-matched analysis of demographic information and patient characteristics between the two groups.

Variable	Patients without cannabis use		Patients with cannabis use		P-value
	N	%	N	%	
N=26,830	13,415	50	13,415	50	
Gender	N	%	N	%	0.205
Female	2400	17.89	2480	18.49	
Male	11,015	82.11	10,935	81.51	
Mean age in years for each gender group	Mean	SD	Mean	SD	
Female	39.14	15.7	40.39	15.49	
Male	36.5	15.92	36.41	15.45	
Age groups in years	N	%	N	%	<0.001
18-29	6020	44.88	5875	43.79	
30-49	3880	28.92	4120	30.71	
50-69	3130	23.33	3150	23.48	
≥70	385	2.87	270	2.01	
Race	N	%	N	%	0.004
Asian or Pacific Islander	140	1.04	155	1.16	
Black	3220	24	3325	24.79	
Hispanic	1270	9.47	1390	10.36	
Native American	100	0.75	110	0.82	
Other	270	2.01	315	2.35	
White	8415	62.73	8120	60.53	
Median household income	N	%	N	%	0.339
≤49,999	4940	36.82	5085	37.91	
50K-64,999	3755	27.99	3685	27.47	
65K-85,999	2930	21.84	2880	21.47	
≥86k	1790	13.34	1765	13.16	
Insurance status	N	%	N	%	<0.001
Medicaid	5000	37.27	4790	35.71	
Medicare	1500	11.18	1740	12.97	
No charge	145	1.08	185	1.38	
Other	450	3.35	395	2.94	
Private insurance	4245	31.64	4165	31.05	

The prevalence of baseline myocardial infarction (p<0.001), obesity (p=0.001), drug abuse (p<0.001), smoking (p<0.001), alcohol consumption (p=0.001), peripheral vascular disease (p=0.001), depression (p<0.001), AIDS (p=0.015), and dementia (p<0.001) was higher among cannabis users than non-cannabis users. The baseline prevalence of autoimmune diseases was higher among non-cannabis users (p<0.001), and the prevalence of other comorbidities was comparable between the two groups (Table 5).

**Table 5:** Propensity score-matched analysis for baseline comorbidities seen in patients with primary spontaneous pneumothorax between the two groups.

Variable	Without cannabis use		Cannabis user		P-value
Comorbidities	Number	%	Number	%	
Coronary artery disease	935	6.97	915	6.82	0.629
Myocardial infarction	260	1.94	350	2.61	<0.001
Hypertension	3075	22.92	3175	23.67	0.148
Diabetes	810	6.04	820	6.11	0.798
Cancer	1060	7.9	1130	8.42	0.118
Obesity	550	4.1	660	4.92	0.001
Drug abuse	975	7.27	6050	45.1	<0.001
Smoking	6400	47.71	9335	69.59	<0.001
Alcohol	1480	11.03	1650	12.3	0.001
Chronic pulmonary disease	5555	41.41	5655	42.15	0.215
Peripheral vascular disease	360	2.68	455	3.39	<0.001
Hypothyroidism	245	1.83	265	1.98	0.371
Autoimmune disease	235	1.75	145	1.08	<0.001
Depression	1290	9.62	1500	11.18	<0.001
AIDS	235	1.75	290	2.16	0.015
Dementia	10	0.07	45	0.34	<0.001
Chronic kidney disease	215	1.6	250	1.86	0.101

After PSM, we found that the risk of sudden cardiac arrest (OR: 0.6; 95% CI: 0.4-0.8; p=0.003), requirement for invasive (OR: 0.6; 95% CI: 0.5-0.7; p<0.001) and non-invasive (OR: 0.6; 95% CI: 0.4-0.9; p=0.005), mechanical ventilation, hemodialysis (OR: 0.7; 95% CI: 0.5-1.0; p=0.047), venous thromboembolism (OR: 0.5; 95% CI: 0.4-0.7; p<0.001), hemothorax (OR: 0.7; 95% CI: 0.9-1.0; p=0.047), ventilator-associated pneumonia (OR: 0.5; 95% CI: 0.2-2.0; p=0.037), and tracheostomy (OR: 0.3; 95% CI: 0.2-0.5; p<0.001) was lower among cannabis users than among non-cannabis users. However, the risk of chest tube placement was higher among cannabis users than non-cannabis users (OR: 1.2; 95% CI: 1.1-1.3; p=0.004). Moreover, the risk of vasopressor requirement (OR: 0.7; 95% CI: 0.7-1.0; p=0.054) and development of acute kidney injury (OR: 0.9; 95% CI: 0.8-1.1; p=0.255) were comparable between the two groups (Table 6).

**Table 6:** Propensity score-matched analysis for complications seen in patients with primary spontaneous pneumothorax between the two groups.

Complications	Number	%	Number	%	Odds ratio	95% CI lower limit	95% CI upper limit	P-value
Sudden cardiac arrest	465	3.47	310	2.31	0.60	0.43	0.84	0.003
Vasopressor use	340	2.53	230	1.71	0.68	0.46	1.01	0.054
Acute kidney injury	1710	12.75	1605	11.96	0.9	0.76	1.08	0.255
Invasive mechanical ventilation	2590	19.31	1825	13.6	0.58	0.5	0.68	<0.001
Non-invasive mechanical ventilation	390	2.91	245	1.83	0.59	0.41	0.86	0.005
Hemodialysis	320	2.39	230	1.71	0.67	0.45	1	0.047
Venous thromboembolism	705	5.26	415	3.09	0.54	0.41	0.72	<0.001
Hemothorax	360	2.68	250	1.86	0.69	0.48	0.99	0.047
Ventilator-associated pneumonia	120	0.89	60	0.45	0.47	0.23	0.96	0.037
Chest tube placement	8110	60.45	8585	64.00	1.18	1.05	1.32	0.004
Tracheostomy	650	4.85	235	1.75	0.33	0.24	0.47	<0.001

The risk of mortality remained lower among cannabis users than non-cannabis users after PSM analysis (3.5% vs. 6.5%; OR: 0.4; 95% CI: 0.3-0.5; p<0.001). The p-trend for mortality remained insignificant at 0.734.

After PSM analysis, the adjusted length of stay was 2.3 days lower among cannabis users (p<0.001), and the adjusted mean inflation-adjusted cost was $11,208.5 lower among cannabis users than among non-cannabis users (p<0.001).

## Discussion

Our study reveals three key findings. First, cannabis users with PSP had a lower risk of mortality than non-cannabis users. Second, cannabis users experienced a shorter hospital stay. Third, cannabis users showed reduced risks of various complications, including sudden cardiac arrest, invasive and non-invasive mechanical ventilation, hemodialysis, venous thromboembolism (VTE), hemothorax, ventilator-associated pneumonia, chest tube placement, and tracheostomy.

In this retrospective study, we examined 399,495 individuals admitted to the hospital with PSP between 2016 and 2019. Among them, 13,415 had both PSP and a concurrent diagnosis of cannabis use, whereas 386,080 had PSP without cannabis use. Our findings indicated that individuals with PSP who used cannabis tended to be predominantly male and younger. Several prior investigations have examined sex roles as a contributing factor to the observed sex disparity in cannabis intake. Similar to the patterns observed with other substances, such as alcohol, men tend to report higher rates of cannabis intake than women [12]. Nevertheless, the current trajectory towards the decriminalization of cannabis and the growing presence of female roles in normalizing cannabis consumption have contributed to a narrowing of the disparity. There is data suggesting the presence of gender biases in the choice of cannabis methods of administration, potentially contributing to gender disparities [12]. The available data indicate that there may be a higher sensitivity to cannabis or cannabinoids among females [13]. Additionally, females may have a faster progression to problematic cannabis use than males, as well as more pronounced withdrawal symptoms during periods of abstinence [14]. These factors may eventually contribute to the observed sex differences in cannabis-related outcomes.

Patients with PSP and cannabis were also more likely to have higher rates of drug use, smoking, alcohol consumption, chronic obstructive pulmonary disease (COPD), depression, and AIDS. This trend may stem from reduced risk perception after cannabis use following decriminalization and a belief in cannabis being safer than tobacco [15,16]. Cannabis's role in treating conditions like neuropathic pain and its use in mood regulation might also correlate with these comorbidities [16]. Additionally, the connection between cannabis use and mood disorders, particularly in younger users, suggests a potential for heightened depression risks [16,17]. It has been claimed that a dose-dependent relationship exists within this correlation [17]. This may explain the higher risk of depression in patients with PSP and cannabis users who were also younger than the non-cannabis user comparison cohort.

The key observation was the reduced risk of various complications in cannabis users, including mortality, sudden cardiac arrest, VTE, and ventilator-associated pneumonia. This aspect of our study offers a new perspective in light of the broader discourse on cannabis and respiratory health, including its potential therapeutic applications [15,16]. However, the intricacies of cannabis's impact on respiratory and overall health are not fully understood, and our findings highlight the need for further research in this area [17,18]. The younger age profile of cannabis users, as identified in our study, might contribute to this observed difference, given the general trend of better clinical outcomes in younger patients. While this might suggest a more favorable clinical course, it is imperative to consider other contributing factors, such as differences in health-seeking behaviors or the severity of the condition upon admission. The complexity of these interactions means that our findings should be interpreted cautiously, avoiding the implication of a direct beneficial effect of cannabis.

When contextualized within the existing literature, Underner et al. conducted a systematic literature review focusing on pneumothorax and lung emphysema in cannabis users. They examined 20 studies, including 18 case reports, and found that bullae in the upper lobes were present in both combined cannabis and tobacco smokers and in cannabis-only smokers [19]. The risk of spontaneous pneumothorax was increased in combined smokers but not in cannabis-only smokers. In our study, we adjusted for smoking use to isolate the specific impact of cannabis on pneumothorax outcomes. This approach differentiates our work from previous studies like Underner et al., which suggested a cumulative effect of both tobacco and cannabis on lung health. Our findings, by focusing solely on cannabis use, contribute uniquely to the understanding of its potential risks and effects. While previous research indicates a heightened risk of pneumothorax and lung emphysema in cannabis users, especially when combined with tobacco smoking, our study sheds light on the implications of cannabis uses alone. This distinction is vital in informing both clinical practice and public health policies, particularly in the context of increasing cannabis legalization and usage.

Limitations

Our study's limitations include reliance on the NIS database, which, while robust, carries inherent drawbacks of retrospective data, such as coding inaccuracies and a lack of detailed clinical information, including PSP etiology and specific cannabis usage patterns. Although PSM was used to mitigate confounding factors, residual confounding remains a possibility. Our findings might not apply to non-hospital settings or populations outside the United States. Additional limitations include potential biases in hospital admission criteria and the exclusion of mild PSP cases not requiring hospitalization. Our study also does not explore the different methods of cannabis uptake among patients, such as smoking, edibles, and vaping, which may introduce heterogeneity among the results. Furthermore, the evolving legal and social context of cannabis use during the study period may affect the generalizability of the findings. Despite these challenges, the study offers significant insights into the effects of cannabis on PSP prognosis in hospitalized patients.

Future directions

Our study underscores the importance of conducting further large-scale prospective observational studies to understand the effects of cannabis consumption on the prognosis of PSP. A multi-centric approach, including data from both outpatient and inpatient settings and ideally data from various countries, can enhance the robustness of the evidence regarding the association between cannabis consumption and its effects on PSP.

## Conclusions

Our study makes a significant contribution to understanding the association between cannabis use and PSP outcomes. It reveals correlations suggesting that cannabis users with PSP might experience lower in-hospital mortality and fewer complications compared to non-cannabis users. However, these observations do not establish causation and should be approached with caution. The study highlights the complexity of cannabis's impact on health and the necessity for more nuanced, prospective research to untangle these associations further, especially in varied patient populations and changing legal landscapes.
